# The yin and yang of co-inhibitory receptors: toward anti-tumor immunity without autoimmunity

**DOI:** 10.1038/s41422-020-0277-x

**Published:** 2020-01-23

**Authors:** Alexandra Schnell, Lloyd Bod, Asaf Madi, Vijay K. Kuchroo

**Affiliations:** 1 Evergrande Center for Immunologic Diseases, Harvard Medical School and Brigham and Women’s Hospital, Boston, MA 02115 USA; 2 Department of Pathology, Sackler Faculty of Medicine, Tel-Aviv University, Tel Aviv-Yafo, Israel; 3 Klarman Cell Observatory, Broad Institute of MIT and Harvard, Cambridge, MA 02142 USA

**Keywords:** Tumour immunology, Autoimmunity, Checkpoint signalling, Cancer immunotherapy

## Abstract

Co-inhibitory receptors are important regulators of T-cell function that define the balance between tolerance and autoimmunity. The immune regulatory function of co-inhibitory receptors, including CTLA-4, PD-1, TIM-3, TIGIT, and LAG-3, was first discovered in the setting of autoimmune disease models, in which their blockade or deficiency resulted in induction or exacerbation of the disease. Later on, co-inhibitory receptors on lymphocytes have also been found to influence outcomes in tumor and chronic viral infection settings. These receptors suppress T-cell function in the tumor microenvironment (TME), thereby making the T cells dysfunctional. Based on this observation, blockade of co-inhibitory receptors (also known as checkpoint molecules) has emerged as a successful treatment option for a number of human cancers. However, severe autoimmune-like side effects limit the use of therapeutics that block individual or combinations of co-inhibitory receptors for cancer treatment. In this review we provide an overview of the role of co-inhibitory receptors in autoimmunity and anti-tumor immunity. We then discuss current approaches and future directions to leverage our knowledge of co-inhibitory receptors to target them in tumor immunity without inducing autoimmunity.

## Introduction

T cells constitute a very important and potent effector compartment of the immune system. Therefore, it is critical that T-cell responses are strictly regulated to avoid inappropriate immune responses, such as autoimmune reactions. Central tolerance in the thymus acts as the first control during T-cell development to eliminate autoreactive T-cell clones. The nuclear factor AIRE expressed in medullary thymic epithelial cells facilitates ectopic expression of tissue-restricted antigens in the thymus and thereby plays an important role in the negative selection of autoreactive T cells in the thymus.^1,2^ The striking autoimmune phenotype in AIRE-deficient mice indicates a dominant role for central tolerance in eliminating autoreactive T cells and thus preventing autoimmune reactions. However, in part due to lack of self-tissue antigen expression in the thymus, altered expression of self-antigens, or low affinity expression of self-antigens, some autoreactive T cells still manage to escape negative selection, leave the thymus and enter the peripheral immune repertoire.^3^ Hence, peripheral regulation of T-cell responses is crucial to prevent inappropriate responses to self-antigens. In the scope of this review we will focus on the role of T cell co-inhibitory molecules in the regulation of peripheral tolerance and autoimmunity, and their role in anti-tumor immunity.

## Co-stimulatory and co-inhibitory receptors

The activation of naïve T cells requires both the stimulation of the T-cell receptor (TCR) by a major histocompatibility complex (MHC)-peptide complex (signal 1) and co-stimulatory signaling by co-stimulatory receptors (signal 2) with their corresponding ligands on antigen-presenting cells (APCs).^4–6^ T cell co-signaling receptors are broadly defined as cell-surface receptors that positively (co-stimulatory) or negatively (co-inhibitory) regulate TCR driven signals and therefore T-cell activation.^6^ As T cell co-signaling receptors have a key role in T-cell biology by directing T-cell activation, expansion and differentiation and therefore T-cell fate, the expression of these co-receptors and their ligands are strictly regulated in T cells and in the tissue micro-environment. An important example of a co-stimulatory pathway is the CD28:B7 axis. The co-stimulatory receptor CD28 on T cells and its ligand B7-1 or B7-2 on activated APCs amplify TCR signaling, leading to T-cell proliferation and IL-2 production.^6,7^ To date, a number of co-stimulatory receptors have been identified including ICOS, CD226, OX-40, 4-1BB, and GITR.^6^ As T cells are being activated and expanded, the expression of co-inhibitory receptors is upregulated. Multiple co-inhibitory receptors have been identified including CTLA-4, PD-1, TIM-3, TIGIT, and LAG-3. Co-inhibitory receptors play an important role in several T-cell subsets including activated T cells, regulatory T cells, and exhausted T cells. In activated T cells, co-inhibitory receptors control and contract the expanded T-cell population. In regulatory T cells (Tregs), co-inhibitory receptors, such as CTLA-4 and PD-1, promote the suppressive function of Tregs.^8,9^ In the scope of this review, we are going to focus on the role of co-inhibitory receptors on exhausted T cells. Recent work identified a critical role of T-cell exhaustion in autoimmune diseases and the targeting of co-inhibitory receptors in cancer therapy has been shown to be limited due to the development of autoimmune-like immune-related adverse events (irAEs). We are therefore interested in discussing the function of co-inhibitory receptors on exhausted T cells in autoimmunity versus anti-tumor immunity and leverage the recent knowledge to improve immune checkpoint blockade therapy for cancer by avoiding the induction of autoimmunity.

## T-cell exhaustion

T-cell exhaustion was originally discovered more than two decades ago, with the observation that virus-specific CD8^+^ T cells from mice with chronic LCMV infections lost the ability to produce effector cytokines and to mediate cytolytic effector functions.^10^ Loss of function during T-cell exhaustion occurs in a hierarchical manner over the course of chronic infection, with loss of both production of IL-2 and T-cell proliferation occurring early after infection.^11,12^ At later stages of T-cell exhaustion, virus-specific CD8^+^ T cells lose the ability to produce the cytokines IFNγ and TNFα, and to degranulate.^11,13^ An additional key property of exhausted CD8^+^ T cells is an impaired maintenance of T-cell memory, which is controlled by the expression of Foxo1.^14^ In contrast to memory CD8^+^ T cells during viral infections, exhausted CD8^+^ T cells respond poorly to the cytokines IL-7 and IL-15 and are not maintained after transfer into virus-free recipient mice.^13,15,16^ Although best characterized in CD8^+^ T cells, CD4^+^ T cells also develop T-cell exhaustion and may play a crucial part in promoting and sustaining T-cell exhaustion in CD8^+^ T cells.^17–20^ Genomic methods including RNA-sequencing and ATAC-sequencing have been used to analyze the molecular pathways underlying T-cell exhaustion.^21,22^ These approaches allowed the identification of a molecular phenotype specific for exhausted T cells, characterizing them as a distinct state of T-cell differentiation, which differs from T-cell activation or memory. The exhaustion-specific molecular changes observed in CD8^+^ T cells include altered metabolism, chemokine and chemokine receptor expression, and cytokine signaling pathways.^21,22^ In addition, a well-defined characteristic of exhausted CD4^+^ and CD8^+^ T cells from both human and animal models is an elevated expression of co-inhibitory receptors.^13,22^ Functional studies have shown an important role for inhibitory receptors in T-cell exhaustion. For example, signaling of the inhibitory receptor PD-1 following binding to its ligand PD-L1 induces T-cell exhaustion, and blocking this pathway during chronic LCMV infection restores virus-specific CD8^+^ T-cell responses by inducing proliferation and cytokine secretion and reduction of viral load.^23–25^ Furthermore, blockade of the PD-1 pathway during chronic simian immunodeficiency virus infection induces rapid expansion of virus-specific CD8^+^ T cells with enhanced effector function resulting in improved survival.^26^

**Fig. 1:**
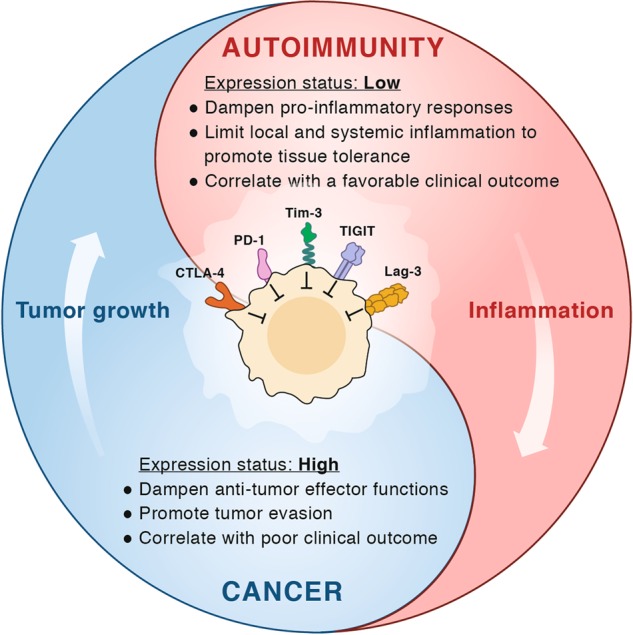
The Yin and Yang of co-inhibitory receptors. Schematic representation of the co-inhibitory receptors’ functional role in autoimmunity and cancer. In the tumor, co-inhibitory receptors on T cells dampen T-cell effector functions thereby enhancing tumor progression and correlating with worse clinical outcome. In autoimmunity, these receptors play a role in reducing local and systemic tissue inflammation, maintaining tissue tolerance, and their increased expression is associated with a good clinical outcome

Importantly, exhaustion has been associated with the clinical outcomes for multiple human diseases. Exhaustion correlates with persistent viraemia in a number of chronic viral infections including human immunodeficiency virus (HIV), hepatitis C and B virus (HCV and HBV).^27–29^ Interestingly, T-cell exhaustion also plays an important role in cancer and autoimmunity, albeit in opposite ways, in that T-cell exhaustion has been correlated with poor immune responses to tumors in patients and with a better prognosis in patients with autoimmune diseases^30,31^ (Fig. 1).

## T-cell exhaustion in autoimmunity

### T cells in autoimmunity

IFNγ-producing CD4^+^ T cells (Th1) and IL-17-producing CD4^+^ T cells (Th17 cells) have specifically been implicated in the development of organ-specific autoimmunity.^32^ Originally several observations supported Th1 cells as the pathogenic subset in T cell-driven autoimmune diseases. Adoptive transfer of Th1 cells induces experimental autoimmune encephalomyelitis (EAE) with the Th1 cytokine IFNγ being present in CNS lesions during EAE peak and decreased during disease remission.^33–35^ Mice deficient for the major Th1 transcription factors, T-bet and STAT-4, show resistance to EAE.^36,37^ Furthermore, administration of the Th1-differentiating cytokine IL-12 aggravates collagen-induced arthritis (CIA).^38^ However, loss of the Th1-signature cytokine IFNγ or STAT-1, the transcription factor that mediates IFNγ signaling, did not inhibit the development of autoimmunity, but paradoxically enhanced autoimmune disease.^36,39,40^ This led to the hypothesis that there might be another subset of T cells driving the development of autoimmunity and tissue inflammation. As IL-17 was found to be increased in mouse and human organ-specific autoimmune diseases, including multiple sclerosis (MS), rheumatoid arthritis, and psoriasis, research focused on the role of IL-17 producing T cells (named Th17 cells) as potential drivers of autoimmunity.^41–44^ Indeed, we and others have shown a potent role for Th17 cells in tissue inflammation and autoimmunity in multiple disease contexts.^45^ Th17 cells were shown to be differentiated in the presence of TGFβ and IL-6 and further expanded or maintained by the cytokines IL-1 and IL-23.^46–48^ Loss of any of the cytokines (IL-6, IL-1, IL-23) resulted in inhibition of the development of autoimmunity.^49–51^ Moreover, differentiation of Th17 cells in the presence of IL-23 induces more severe EAE.^48^ It is important to note, however, that Th1 and Th17 cells are non-mutually exclusive cell fates. IFNγ and IL-17 double-positive cells have been observed in the CNS during EAE and fate mapping of IL-17 producing T cells has revealed Th17 cell plasticity to an IFNγ^+^ Th1-like phenotype.^52–55^ Future studies are needed to further elucidate the function of the Th17/Th1 plasticity in the pathogenesis of autoimmune diseases.

Understanding the role of T cells in autoimmunity has led to the successful development of agents for the treatment of organ-specific autoimmune diseases. Monoclonal antibodies (mAbs) blocking IL-17A, secukinumab and ixekizumab, are approved for the treatment of psoriasis and secukinumab is also approved for the treatment of psoriatic arthritis and spondyloarthropathies.^56^ Similarly, drugs targeting the proinflammatory cytokine TNFα (infliximab, etanercept, adalimumab) have been approved for the treatment of multiple human autoimmune diseases including rheumatoid arthritis, Crohn’s disease, and ankylosing spondylitis.^57^

However, current therapies targeting cytokines and/or cytokine signaling are not efficacious in all patients with autoimmune disease. Rather each agent must be tested in each disease, and typically not all patients diagnosed with a particular disease respond to treatment. Therefore, there is a need for the development of novel therapeutic approaches that will impact a common critical point that will enable efficacy in more patients and across diverse autoimmune diseases. One potential approach is to promote T-cell exhaustion by regulating expression or function of “checkpoint” molecules in autoimmune diseases.

### T-cell exhaustion in autoimmunity

To identify predictive biomarkers linked to clinical outcome and novel therapeutic targets in autoimmune patients, McKinney et al. undertook an unbiased genomic analysis of CD4^+^ and CD8^+^ T cells from patients suffering from various autoimmune diseases.^31^ The authors demonstrated that the level of T-cell exhaustion in autoimmune patients was strongly correlated with their clinical outcomes. Using independent cohorts of patients suffering from the autoimmune diseases anti-neutrophil cytoplasmic antibody-associated vasculitis (AAV), SLE, and inflammatory bowel disease (IBD), they found that for all three diseases, the T-cell exhaustion signature predicted a favorable clinical outcome. Hence, whereas T-cell exhaustion during chronic viral infections correlates with persistent viremia and worse clinical outcome, in autoimmune diseases the effect is opposite in that T-cell exhaustion seems to predict a better clinical outcome.

**Fig. 2:**
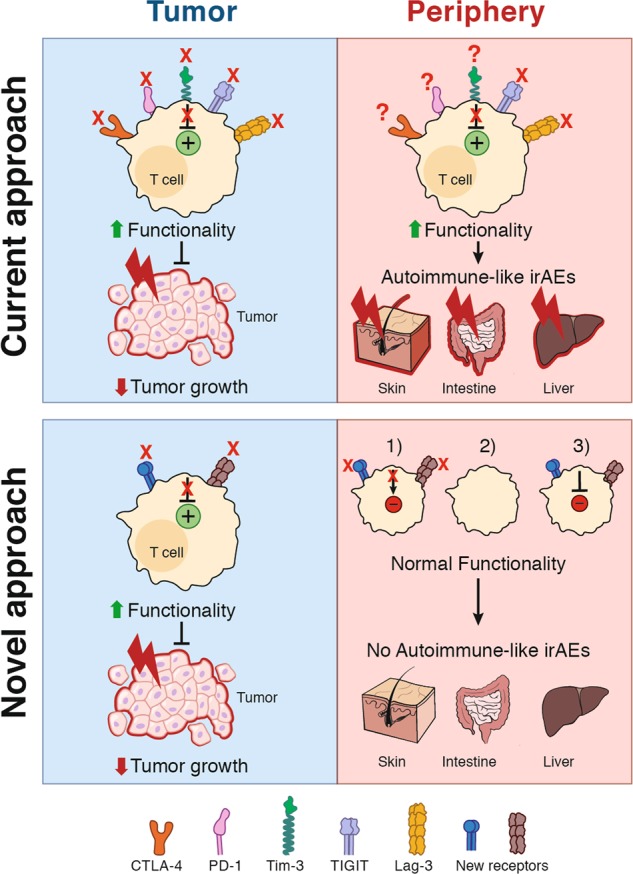
Novel approaches for checkpoint blockade therapy to avoid autoimmune-like disease. Top: Current immune checkpoint blockade inhibits the signaling of co-inhibitory receptors thereby enhancing T-cell effector functions. At the tumor site, these hyper-functional T cells mediate strong anti-tumor immunity thereby reducing tumor growth (left). However, the T cells with specificity for self-antigens induce severe autoimmune-like irAEs by becoming hyper-functional and inducing tissue inflammation (right). IrAEs are mostly found in tissues with high microbial exposure, such as the skin, intestine, and liver. At homeostasis these tissues manifest a well-regulated tolerogenic environment, that is disrupted with checkpoint blockade. Bottom: Novel approaches should target new receptors mediating a potent immune response in the tumor (left) while reframing from inappropriate immune responses in the periphery against self-antigens (right). Potential mechanisms are: (1) in the periphery and in normal tissues the receptors act differentially, (2) the receptors are not expressed in the periphery, (3) the blockade only occurs in the tumor and not in the peripheral tissues

Additional data suggesting an important role for T-cell exhaustion in maintaining tolerance and preventing of autoimmunity has recently emerged following blockade of co-inhibitory molecules to promote anti-tumor immunity. The blockade of PD-1 and CTLA-4 with mAbs in cancer patients often leads to severe irAEs in multiple tissues, including the skin, intestine, liver and lung^58,59^ (Fig. 2). These irAEs present pathologically like autoimmune diseases, and therefore support the concept that co-inhibitory receptors maintain peripheral tolerance, thereby preventing inappropriate autoimmune tissue inflammation.

Whereas the link between T-cell exhaustion and clinical outcomes in human autoimmunity has emerged only recently, the role of co-inhibitory molecules in autoimmune disorders has long been appreciated. In the following part of this review we will summarize the main findings highlighting the role of the co-inhibitory receptors CTLA-4, PD-1, TIM-3, TIGIT, and LAG-3 in autoimmune pathology.

### Co-inhibitory receptors on T cells in autoimmunity: a focus on CTLA-4, PD-1, TIM-3, TIGIT and LAG-3

The study of T cell co-inhibitory receptors began with the discovery of cytotoxic T lymphocyte-associated antigen-4 (CTLA-4, CD152) in 1987 as a receptor for B7 with high sequence similarity to CD28.^60,61^ However in contrast to CD28, CTLA-4 was found to act as a negative regulator of T-cell activation by binding to the same ligands as the co-stimulatory molecule CD28, B7-1 and B7-2, but with much higher affinity.^62–64^ Blockade of CTLA-4 in vitro and in vivo enhanced T-cell proliferation and effector functions.^65,66^ The subsequent generation of CTLA-4-deficient mice set the stage for the discovery of the important inhibitory function of CTLA-4 in autoimmunity. Mice with germline deletion of CTLA-4 developed lethal autoimmunity characterized by lymphoblast infiltration into liver, heart, lung, and pancreas and death by 3 to 4 weeks of age.^67,68^ Deletion of CTLA-4 in adult mice led to spontaneous lymphoproliferation and non-lethal autoimmune disease in multiple organs.^69^ Moreover, in vivo blockade of CTLA-4 signaling was shown to exacerbate disease in multiple murine autoimmune models.^70^ For example, administration of blocking anti-CTLA-4 mAb into young diabetes-susceptible mice (BDC2.5/NOD mice) provoked rapid onset of diabetes.^71^ Furthermore, in relapsing-remitting EAE, treatment with anti-CTLA-4 mAbs enhanced disease.^72^ However, it is important to note that in contrast to the germline deletion, CTLA-4 deficiency in adulthood leads to the protection from EAE.^69,73^ These paradoxical observations can be explained by compensatory immunosuppressive mechanisms following CTLA-4 deletion and an inhibitory role of CTLA-4 on Treg cells. Deletion of CTLA-4 in adult T cells led to the upregulation of inhibitory molecules including IL-10, LAG-3, and PD-1. Furthermore, the deletion of CTLA-4 specifically on Treg cells was necessary and sufficient to mediate EAE protection.^73^

In humans, in addition to the full-length form of CTLA-4 (flCTLA-4), a soluble form of CTLA-4 exists (sCTLA-4) that lacks the transmembrane domain encoded by exon 3.^74,75^ Interestingly, sCTLA-4 has been associated with type 1 diabetes (T1D). Indeed, T1D disease susceptibility mapped to an allelic variation in the 3’ noncoding region of CTLA-4 correlates with mRNA expression of sCTLA-4.^75^ However, in NOD mice, a mouse model for T1D, disease susceptibility correlates with differential expression of a different CTLA-4 splice variant, ligand-independent CTLA-4 (liCTLA-4), that lacks the B7-1/B7-2 binding domain.^76,77^ LiCTLA-4 was shown to be a potent inhibitor of T-cell proliferation and cytokine secretion.^78^ These different variants might occur differentially depending on the autoimmune settings, affecting the tolerance mechanisms involved.

In 1992, the second co-inhibitory receptor programmed death-1 (PD-1, CD279) was discovered and was shown to express both ITIM and ITSM signaling motifs.^79^ Like CTLA-4, PD1 is an inhibitory receptor on T cells that mediates its inhibitory signals via its ligands PD-L1 (B7-H1, CD274) and PD-L2 (B7-DC, CD273). PD-1 is expressed on CD4^+^ and CD8^+^ T cells, B cells, monocytes, and subsets of dendritic cells (DCs).^80^ Whereas PD-L1 is broadly expressed on hematopoietic and non-hematopoietic parenchymal tissue cells, PD-L2 is only expressed on DCs and some subsets of myeloid cells. Studying PD-1 deficiency in autoimmune disease mouse models has elucidated an important role for the PD-1 pathway in autoimmune diseases. Germline deletion of PD-1 results in development of severe autoimmune disease. Whereas BALB/c PD-1^−/−^ mice develop lethal dilated cardiomyopathy, deletion of PD-1 in C57BL/6 mice results in spontaneous lupus-like autoimmune disease.^81,82^ T1D-prone NOD mice that are deficient for PD-1 have accelerated diabetes onset and an increased incidence of diabetes.^83^ Furthermore, blockade of PD-1 in EAE results in accelerated and more severe disease progression, with an increased infiltration of mononuclear cells into the CNS.^84^ Polymorphisms in the PD-1 locus in humans have been associated with SLE, T1D, ankylosing spondylitis, and rheumatoid arthritis.^85–88^

The co-inhibitory receptor T cell immunoglobulin and mucin-domain containing protein-3 (TIM-3) was originally discovered in our laboratory as a surface protein specifically expressed on Th1 cells and IFNγ-producing CD8^+^ T cells.^89^ As Th1 cells are established drivers of autoimmune disease, the function of TIM-3 was first studied in models of autoimmunity and it became apparent that TIM-3 mediates a potent inhibitory function. TIM-3 is expressed on CD4^+^ and CD8^+^ T cells, NK cells, and myeloid cells, such as DCs and monocytes.^90^ The C-type lectin galectin-9 was the first ligand identified for TIM-3.^91^ Subsequently, several other ligands of TIM-3 have been identified, including phosphatidylserine and CEACAM1.^92,93^ Administration of anti-TIM-3 antibodies in EAE results in enhanced clinical and pathological disease scores with an increased activation phenotype in macrophages.^89^ Blockade of TIM-3 signaling in 2,4,6-trinitrobenzene sulfonic acid (TNBS)-induced colitis exacerbates disease, as shown by enhanced weight loss and tissue injury.^94^ Moreover, TIM-3 pathway blockade was observed to accelerate autoimmune diabetes.^95^ In addition to the results from murine autoimmune models, multiple studies also indicated an important role for TIM-3 in human autoimmune diseases. Hafler and colleagues first showed that T-cell clones generated from the cerebrospinal fluid (CSF) of MS patients produce high levels of IFNγ but show decreased expression of TIM-3 compared to clones from control subjects.^96^ Interestingly, treatment with IFNβ, an FDA-approved drug for MS, restored TIM-3 expression and lessened disease activity.^97^ Similarly, a number of studies have shown that reductions in TIM-3 expression on CD4^+^ and CD8^+^ T cells are inversely correlated with disease activity in patients with other autoimmune diseases, including rheumatoid arthritis, ulcerative colitis and psoriasis.^98–100^ In rheumatoid arthritis patients, TIM-3 expression is inversely correlated with disease activity and plasma TNFα levels. Following treatment, TIM-3 expression increases and is associated with disease remission. In addition to Th1 cells, Th17 cells have been shown to express TIM-3, but at a lower level than on Th1 cells.^101^ In patients with psoriasis, TIM-3-negative Th1 and Th17 cells are increased in the peripheral blood, suggesting that impaired TIM-3 expression allows Th17 and Th1 cells to escape from TIM-3-mediated immunoregulation and thereby mediate disease.^98^ Together, these studies indicate that manipulating TIM-3 signaling in vivo may be a valuable tool in the treatment of autoimmunity. In addition to these polygenic autoimmune diseases, a recent study identified germline TIM-3 mutations in patients with subcutaneous panniculitis-like T-cell lymphoma (SPTCL).^102^ The mutations lead to misfolding of TIM-3 so that the protein is retained intracellularly with loss of cell surface expression. Patients harboring such loss of function mutations present with a severe autoinflammatory disease with high levels of IL-1 and TNFα in the serum and CD8^+^ T cells forming lymphomas around subcutaneous fat pads. About 30% of these patients develop autoimmune lupus-like disease characterized by elevated production of antibodies directed to double-stranded DNA. Furthermore, polymorphisms in the TIM-3 locus have been associated with multiple human autoimmune disorders.^103^

Another co-inhibitory molecule, T cell immunoglobulin and ITIM domain (TIGIT) was discovered as a novel member of the CD28 protein family that is specifically expressed on immune cells. TIGIT is expressed on activated T cells, a subset of Treg cells and Tfh cells, and NK cells.^90,104–107^ TIGIT signals via two ligands, CD155 (PVR) and CD112 (PVRL2), that are expressed on APCs, T cells, and some non-hematopoietic cells.^90,106,107^ Interestingly, in tumors (murine and human) both immune cells and tumor cells express high levels of TIGIT ligands. The biological function of TIGIT was initially investigated in models of autoimmunity. Mice deficient for TIGIT did not display spontaneous autoimmunity. However, the loss of TIGIT led to exacerbated autoimmune disease after immunization, or when TIGIT-deficient mice were crossed to an appropriate auto-reactive T-cell mouse line. TIGIT-deficient mice are highly susceptible to actively-induced EAE with enhanced T-cell infiltration into the CNS and increased pro-inflammatory cytokine levels.^108^ Furthermore, TIGIT-deficient mice crossed to the 2D2 MOG-specific TCR transgenic mice display spontaneous atypical EAE.^108^ Additionally, TIGIT was also found to have a protective function in CIA. Administration of TIGIT-blocking antibodies resulted in accelerated disease onset of CIA.^109^ Collectively, these data suggest an inhibitory function of TIGIT on T cells in murine autoimmune models. CD226 binds to the same ligands as TIGIT, but in contrast to TIGIT, mediates a positive co-stimulatory signal, forming a network similar to the B7:CD28:CTLA4 co-stimulatory molecules. Interestingly, genome-wide association studies have linked a polymorphism in CD226 to multiple human autoimmune diseases including MS and T1D, suggesting that the TIGIT:CD226 pathway may also play a role in human autoimmunity.^110,111^

The co-inhibitory receptor lymphocyte activation gene-3 (LAG-3) was discovered as a receptor expressed on activated T cells and a subset of NK cells.^112^ Interestingly, the structure of LAG-3 resembles the CD4 receptor and, in fact, LAG-3 binds to MHC class II with a higher affinity than CD4.^113^ In addition to MHC class II, the DC-SIGN family member LSECtin and the liver-secreted protein FGL1 have been identified as ligands of LAG-3.^114,115^ In contrast to mice deficient in other co-inhibitory receptors, such as CTLA-4 and PD-1, mice deficient for LAG-3 are not susceptible to autoimmunity unless bred to a permissive genetic background. LAG-3 deficiency in B6.SJL mice results in higher susceptibility to Hg-induced autoimmunity.^116^ In addition, NOD mice deficient for LAG-3 display accelerated T1D with 100% incidence.^117^ Interestingly, the NOD LAG3^−/−^ mice exhibit increased T-cell numbers and enhanced proliferation of T cells in the islets, suggesting an inhibitory role for LAG-3 on T cells.

Understanding how co-inhibitory receptors are individually and collectively induced and involved in the establishment of the T-cell exhaustion state in autoimmunity would be a corner stone for their manipulation in the clinic.

### Therapeutic induction of exhaustion in autoimmune diseases

**Fig. 3:**
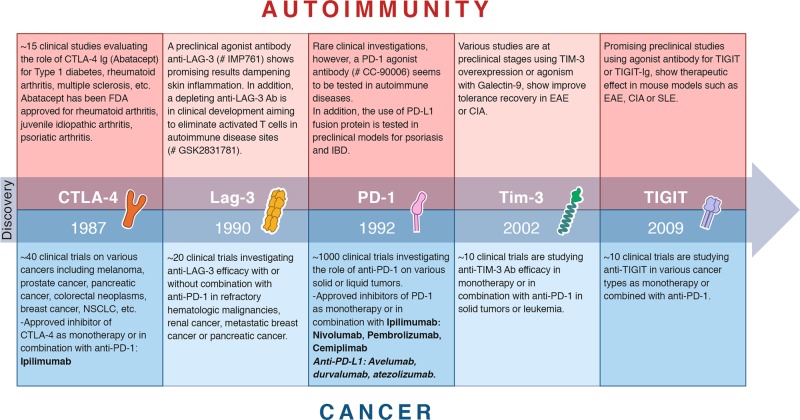
Therapeutic targeting of co-inhibitory receptors in autoimmunity and cancer. Timeline of discovery and therapeutic targeting of the five co-inhibitory receptors discussed in this review: CTLA-4,^60^ LAG-3,^112^ PD-1,^79^ TIM-3,^89^ TIGIT.^106^ The clinical or preclinical investigations and FDA-approved drugs are listed for both autoimmunity (red) and cancer (blue) according to https://clinicaltrials.gov and https://www.fda.gov

As T-cell exhaustion correlates with a poor outcome in cancer patients, there has been a considerable interest in targeting T-cell exhaustion to enhance immune responses to cancer cells.^13^ Targeting T-cell exhaustion using PD-1 blocking antibodies is a proof of concept that blockade of co-inhibitory receptors can restore a functional T-cell response. As mentioned earlier, in contrast to cancer, multiple lines of evidence suggest a beneficial role for T-cell exhaustion in restraining autoimmune responses, in that deficiency or blockade of inhibitory receptors results in the development of autoimmunity in mice, and induction of autoimmunity is observed in humans during immune checkpoint blockade therapy for cancer. Additionally, a transcriptional signature of T-cell exhaustion is associated with a favorable clinical outcome in multiple human autoimmune diseases.^31^ Based on these data, promoting T-cell exhaustion could be beneficial in autoimmunity, and provides a novel therapeutic approach for inhibiting autoimmunity^31,118^ (Fig. 3 and Table 1). Agonist antibodies and Fc-fusion proteins that engage co-inhibitory receptors have been successful in treating murine models of autoimmunity.^119^ For example, cells from the draining lymph nodes of mice treated with agonistic anti-TIGIT antibodies showed reduced cell proliferation and proinflammatory cytokine production after restimulation with MOG-peptide. Moreover, the severity of EAE was reduced in treated mice accompanied by decreased frequencies of IL-17-producing lymphocytes in the CNS.^120^ The administration of PDL-1.Fc to crosslink PD-1 ameliorated the severity of CIA, which was accompanied by decreased proliferation of T cells.^121^ Furthermore, administration of the TIM-3 ligand Galectin-9 decreased CIA severity.^122^ However, these studies did not prove that the agonistic agents directly induce signaling through the co-inhibitory receptors. Hence, future mechanistic studies will be required for translating the use of compounds triggering co-inhibitory receptors for the treatment of autoimmune diseases to the human setting.

**Table 1:** Co-inhibitory receptors in autoimmunity and cancer

Inhibitory receptor	Autoimmunity	Cancer
**CTLA-4** (cytotoxic T lymphocyte-associated antigen-4)	- Global knockout: lethal autoimmunity^67,68^ - Deletion in adult mice: non-lethal autoimmune disease^69^ - In vivo blockade: autoimmune disease exacerbation in multiple murine models (diabetes, EAE, etc.)^71,72^ - Human disease association: multiple including T1D, autoimmune thyroid disease, and rheumatoid arthritis^267–269^	- Inducible knockout: No effect on tumor growth^73^ - Conditional deletion in Tregs: Reduced transplantable tumor growth^9^ - In vivo blockade: Tumor control and/or regression in murine tumor models^66,154,155,224^ - First FDA-approved checkpoint blockade therapy for metastatic melanoma^213^
**PD-1** (programmed death-1)	- Global knockout: Severe autoimmune disease. Phenotype depends on mouse strain: BALB/c lethal dilated cardiomyopathy, C57BL/6 mice lupus-like autoimmune disease, NOD mice exacerbated diabetes^81–83^ - In vivo blockade: Accelerated and more severe EAE^84^ - Human disease association: Multiple including SLE, T1D, ankylosing spondylitis, and rheumatoid arthritis^85–88^	- Global knockout, in vivo blockade or conditional deletion of PD-1 in T cells lead to accelerated tumor clearance in multiple murine cancer models^168–171^ - Anti-PD-1 antibodies induce tumor regression in patients with melanoma, renal cancer, lung cancer, and colon cancer^270^
**TIM-3** (T cell immunoglobulin and mucin-domain containing protein-3)	- Global knockout: Dysregulated Th1 cells in EAE^271^ - In vivo blockade: Exacerbates EAE, TNBS-induced colitis, and diabetes^89,94,95^ - Human disease association: TIM-3 levels decreased in T cells from autoimmune disease patients,^96,98–100^ polymorphisms associated with multiple human autoimmune disorders,^103^ germline TIM-3 mutations in patients with SPTCL^102^	- TIM-3 overexpression on T cells promotes tumor growth^175^ - In vivo blockade of TIM-3 reduces tumor growth^179^ - Promising results in clinical studies on solid tumors, especially in combination with PD-1 blockade^187^
**TIGIT** (T cell immunoglobulin and ITIM domain)	- Global knockout: Highly susceptible to EAE^108^ - In vivo blockade: Accelerated disease onset of CIA^109^ - Human disease association: Polymorphism in CD226 linked to multiple human autoimmune diseases^110,111^	- Global knockout and conditional knockout in Tregs but not CD8^+^ T cells reduce tumor growth in vivo^195^ - In vivo blockade: Synergized with anti-PD-1 treatment leading to tumor regression^120,196^ - Clinical studies showing beneficial therapeutic impact of TIGIT blockade or co-blockade with PD-1 in multiple cancer types^196,201,202^
**LAG-3** (Lymphocyte activation gene-3)	- Global knockout: No increased susceptibility to autoimmune disease unless crossed to permissive genetic background. LAG3-deficient NOD mice accelerated T1D,^117^ LAG3-deficient B6.SJL mice higher susceptibility to Hg-induced autoimmunity^116^	- Global knockout or in vivo blockade reduces the growth of transplantable tumors^203^ - Strong synergistic effect with PD-1 to promote tumor progression^209–212^ - Encouraging clinical trials evaluating LAG-3-targeted therapies in cancer patients^272^

Moreover, in order to facilitate the induction of T-cell exhaustion as therapy for human autoimmune diseases, a better understanding of the underlying biology of T-cell exhaustion is required. In a recent study, our laboratory used unbiased RNA and protein expression profiling to identify a module of co-inhibitory receptors in CD4^+^ and CD8^+^ T cells.^123^ Interestingly, our studies identified the immunoregulatory cytokine IL-27 as a key inducer of the co-inhibitory gene module. In addition, the transcription factors PRDM1 and c-MAF, induced by IL-27, were identified as cooperative regulators of the co-inhibitory gene module. Loss of both PRDM1 and c-MAF resulted in an about 2-fold decrease in expression of the co-inhibitory genes accompanied by increased anti-tumor immunity. Additional studies using novel unbiased computational methods are going to be required in the future to identify and test mediators of T-cell exhaustion in the treatment of autoimmune diseases.^124^ Furthermore, a better analysis of the tissue-specific molecular and cellular pathways inducing T-cell exhaustion remains mandatory in order to improve its targeting.

## T-cell exhaustion in cancer

### T cells in tumor immunity

Historically, cancer research has been dominated by a tumor-centric view, deciphering intrinsic features of tumor cells. However, Ehrlich’s theories, Coley’s clinical observations and experimental studies from Macfarlane Burnet and Lewis Thomas provided a strong rationale for cancer immunosurveillance.^125–129^ In recent years, the concept of tumor cells interacting with the immune system and other cell types has become the focus of a number of studies. Among the cell types composing the TME, T cells have gained the most attention in cancer research. Tumor cells harboring genetic alterations differ from normal cells and are able to induce tumor-reactive T-cell responses.^130–134^ During the T-cell response to cancer, tumor antigen-experienced lymphocytes undergo activation and differentiation into effector and memory fates.^135–137^ Heterogeneity among these two cell fates has been demonstrated, with the description of multiple effector and memory subsets.^137–139^ T cells are present in the TME of most solid tumors and it is well established that infiltration of T cells, particularly CD8^+^ T cells, correlates with a positive clinical outcome in several cancer types.^140,141^ CD4^+^ T cells have the ability to engage various differentiation pathways including the Th1-type pathway that may have a direct anti-tumor role via the secretion of IFNγ or TNFα. However, the most notable effect of CD4^+^ T cells is to provide help to expand and differentiate CD8^+^ T cells into cytotoxic T lymphocytes (CTLs). With the help of DCs, CD8^+^ T cells are able to recognize and lyse tumor cells via the duo granzyme B/perforin, FasL and TRAIL molecules. However, as described above in chronic infections and cancer, a significant fraction of antigen-specific CD8^+^ T cells attain a hypo-responsive state called T-cell “exhaustion”.

### T-cell exhaustion in tumor immunity

T-cell exhaustion was a term first coined in the context of chronic viral infection, as a state of dysfunctional phenotype where T cells progressively lose their effector function due to chronic antigen exposure, further promoted by a lack of CD4^+^ T-cell help and exposure to immunosuppressive cytokines.^22,142^ This concept expanded to the field of cancer research due to high tumor-antigen loads and the tolerogenic TME. T cells isolated from human tumors or murine models were initially described as phenotypically and functionally similar to exhausted T cells described in chronic infections, however  there might be nuanced differences between those exhausted T cells found in chronic viral infections and the ones found in the TME.^143,144^ However, for the purpose of this review the dysfunctional T cells found in TME will be referred to as exhausted T cells. Exhausted T cells are characterized as a fraction of tumor-reactive CD8^+^ T cells that are unable to lyse tumor cells, and have impaired effector functions including production of potent effector cytokines (e.g., TNFα, IFNγ, IL-2) together with high expression of co-inhibitory receptors including CTLA-4, PD-1, TIM-3, TIGIT or LAG-3.^13,21,22,145^ More recently, there has been an increasing focus on the transcriptional regulators that initiate, amplify or maintain the exhaustion state. Singer et al. have identified different gene programs regulating CD8^+^ tumor-infiltrating lymphocytes (TILs) dysfunction. The zinc-finger transcription factor GATA-3 was highlighted to be one of the drivers of the dysfunctional state in those cells.^123,145^ A recent publication by Vodnala et al. showed that the TME nutrients and metabolites milieu, especially potassium concentrations, impact the T-cell genome and epigenome, triggering T-cell stemness.^146^ Moreover, the exhaustion-fate commitment of CD8^+^ T cells has been recently attributed to the expression of the HMG-box transcription factor TOX, which appears to be critical for initiating the exhaustion epigenetic program in both chronic viral infections and cancer.^147–149^ Dissecting the exhaustion state and transcriptional program remains essential in order to improve the identification and targeting of exhausted T cells in cancer. Expression of inhibitory receptors present on the surface of exhausted T cells form a functional module which is tightly controlled within the TME.^123,145^ In line with the previous section regarding the role of checkpoint receptors in autoimmunity, we will explore several inhibitory receptors and will highlight rationale, mechanisms and recent advances from both experimental models and clinical data emphasizing the value of those receptors as potential targets to manipulate for cancer therapy.

### Co-inhibitory receptors on T cells in anti-tumor immunity: a focus on CTLA-4, PD-1, TIM-3, TIGIT and LAG-3

Promptly after their discovery, investigations aiming to target co-inhibitory receptors arose and established a new paradigm in therapies for cancer. As previously described, CTLA-4 acts as a brake on T-cell responses. In cancer, CTLA-4 is expressed on activated CD4^+^ and CD8^+^ T cells and on Treg cells.^9,150,151^ CTLA-4 can also be expressed by tumor cells themselves.^152^ Unfortunately, the rapid and fatal autoimmunity in CTLA-4-deficient mice limits the analysis of experimental tumor models.^67,68^ Interestingly, the inducible deletion of CTLA-4 expression in adult mice has no effect on the growth of MC38 colon adenocarcinoma cells in vivo, raising questions regarding the mechanisms involved in CTLA-4 targeting therapies.^73^ The first evidence that fostered enthusiasm in the field was the in vivo blockade of CTLA-4, published in 1996, leading to a remarkable control of tumor growth in mice.^66^ Notably, anti-CTLA-4 treatment restored exhausted T-cell effector functions, but the therapeutic effect was mainly due to the Fc-dependent depletion of Tregs occurring after anti-CTLA-4 administration.^153–156^ Moreover, conditional deletion of CTLA-4 specifically in Tregs revealed important insights into their ability to promote tumor growth.^9^ In human cancer, several studies have described a negative correlation between the levels of CTLA-4 mRNA or protein and the clinical outcome in both leukemic and solid tumors.^157–159^ Interestingly, in non-small cell lung cancer (NSCLC), high CTLA-4 expression in primary tumors predicts an improved patient survival, whereas its expression in the sentinel lymph node correlates with a poor clinical outcome.^160^ Multiple studies identified SNPs within the CTLA-4 locus associated with various effects on cancer outcome.^161^ In particular, some SNPs in the promoter region of the CTLA-4 locus that regulate its expression, have been associated with higher susceptibility to pancreatic cancer or breast cancer.^162,163^ These data pave the way for improved personalized medicine with a deeper screening of these variants allowing the adjustment of targeted therapies.

PD-1 was initially identified as a receptor on T cells associated with programmed cell death.^79^ Further studies led to evidence for its role in anti-tumor immune responses. Its ligands, PD-L1 and PD-L2 expressed on various tumors and myeloid cells, dampen anti-tumor immune responses.^164–166^ Interestingly, tumor-derived PD-L1 is sufficient to directly inhibit CD8^+^ T cells, thereby promoting tumor escape.^167^ Moreover, blockade, germline knockout or specific deletion of PD-1 in T cells leads to accelerated tumor clearance in multiple mouse models.^168–171^ In several types of cancer, the expression of PD-1 and/or its ligands is elevated and has been associated with poor prognosis and is predictive of response to antibodies targeting the PD-1 pathway.^172,173^ Several studies have also identified PD-1 polymorphisms and their association with susceptibility to various cancer types.^174^ These studies have led to the successful therapeutic targeting of CTLA-4 and PD-1 for various malignancies.

Beyond these two receptors, preclinical data support a role for three other co-inhibitory receptors, TIM-3, TIGIT, and LAG-3, in cancer. Expression and function of TIM-3 was rapidly deciphered in cancer biology, due to its role in the regulation of type 1 immune responses.^89,91,175–177^ TIM-3 was found to be highly expressed by CD8^+^ PD-1^+^ T cells which include the most dysfunctional subset of tumor-infiltrating lymphocytes in tumors.^178,179^ TIM-3 overexpression on T cells leads to the expansion of myeloid-derived suppressor cells and promotes tumor growth.^175^ In contrast, the blockade of TIM-3 reduces tumor growth and ameliorates tumor-specific CD8^+^ T-cell responses.^179,180^ High TIM-3 expression was shown to be associated with a poor prognosis in multiple human cancers such as colon, gastric, non-small cell lung, and clear cell renal carcinoma.^181^ In human colorectal cancer patients, high TIM-3 concentration correlates with disease progression.^182^ Several TIM-3 polymorphisms have been reported to be associated with a higher risk of digestive cancer, renal cell carcinoma, pancreatic cancer and non-small cell lung cancer.^183–186^ At least 9 different clinical trials investigating TIM-3 inhibition in solid tumors show promising results, especially in combination with PD-1 blockade^187^(Clintrials.gov).

TIGIT is highly expressed on NK cells, CD8^+^ T cells and Treg cells within the TME.^188–191^ Its ligands CD155 and CD112 are widely expressed on tumor cells.^192–194^ Multiple studies showed that TIGIT signaling is involved in dampening both T-cell activation and differentiation into anti-tumor effector cells.^107^ Interestingly, the majority of tumor-infiltrating Tregs expresses TIGIT, and TIGIT-deficient Tregs, but not CD8^+^ T cells, are able to reduce tumor growth in vivo.^195^ More recently, blocking anti-TIGIT antibodies synergized with anti-PD-1 treatment leading to tumor regression in vivo.^120,196^ In cancer patients, TIGIT expression has been associated with a poor clinical outcome in liquid or solid tumors.^197–200^ Currently, a number of clinical studies are investigating the therapeutic impact of TIGIT blockade either alone or in combination with PD-1 in multiple cancer types.^196,201,202^

The checkpoint receptor LAG-3 holds great potential in cancer immunotherapy. LAG-3 is expressed widely in tumor-infiltrating lymphocytes in various tumors.^203–205^ Initially described to modulate anti-tumor cell cytotoxicity in vitro,^205–208^ the blockade or germline deletion of LAG-3 reduces the growth of transplantable tumors in vivo.^203^ Remarkably, LAG-3 synergizes strongly with PD-1 to promote tumor progression.^209–212^ Ongoing investigations are highlighting new ligands for LAG-3 (e.g., galectin-3 or Fibrinogen-like protein 1) that are present in the TME together with novel immunomodulatory functions that will consolidate the basis for future therapeutic development of LAG-3 targeting reagents. Particularly, the high affinity interaction of Fibrinogen-like protein 1 (FGL1) and LAG-3 has revealed a major immune-evasion mechanism suppressing T cell anti-tumor responses and may constitute an important targetable pathway in cancer immunotherapy.^115^ A comprehensive appreciation of the receptor-ligand interactions and their biology remains essential for an optimal therapeutic targeting in clinic.

### Manipulating T-cell exhaustion in cancer

#### Checkpoint blockade-based immunotherapy

Based on initial studies on mouse tumors, anti-CTLA-4 antibodies were tested in clinical trials for human cancers. In 2010, targeting CTLA-4 pioneered checkpoint blockade-based immunotherapy by improving the survival of patients with metastatic melanoma.^213^ A year after the publication of the clinical trial, anti-CTLA-4 immunotherapy was approved by the FDA for advanced melanoma, paving the way for targeting of other checkpoint receptors such as PD-1 in 2014. However, the response rate of patients to these monotherapies remained modest. For example, in patients with metastatic melanoma, the objective response rate (ORR) for ipilimumab (anti-CTLA-4) was about 10%–16% and for nivolumab (anti-PD-1) was 30%–40%. Combinations of agents targeting CTLA-4 and PD-1 were thus investigated to increase the ORR and survival rates of patients. Indeed, in several studies, combination of ipilimumab and nivolumab increased the ORR to about 60% in various cancers,^214–218^ reaching 90% ORR in melanoma.^214,219^ However, some tumors were refractory or acquired tumor-resistance to these therapies preventing clinical success.^220^ Furthermore, a significant fraction of patients developed severe irAEs, especially with combined CTLA-4 and PD-1 blockade, increasing the clinical need to optimize these therapies.^221^ Multiple investigations of optimized therapies (combination with other therapies, treatment kinetics, specificity, various Ig-isotypes or Fc-engineered antibodies) aim to improve the efficacy while mitigating the toxicity of anti-PD1 and anti-CTLA-4 antibodies.^222–224^ Interestingly, a recent study showed that in combination with vaccines against tumor-associated antigens, the timing of anti-PD-1 blockade influences therapeutic outcome. Particularly, PD-1 blockade under suboptimal priming conditions leads to the emergence of a subset of dysfunctional CD8^+^ T cells that prevent antitumor immunity.^225^ However, these observations have catalyzed exploration into novel co-inhibitory molecules expressed on exhausted T cells with the goal of identifying receptors with more tumor-restricted expression to achieve increased efficacy while limiting autoimmune-like toxicity. Multiple clinical trials are currently investigating the use of TIM-3, TIGIT and LAG-3 as targets for monotherapy or in combination with anti-PD-1 and have shown promise for cancer therapy^90,217,218,226^ (Fig. 3 and Table 1).

#### Checkpoint-inhibitor induced immune-related adverse events (irAEs)

As mentioned above, perturbing immune exhaustion or tolerance can provoke inappropriate autoimmune reactions (Fig. 2). Following checkpoint blockade, these overexuberant responses lead to irAEs, which could derive from: 1. an aggravation of a silent pre-existing autoimmune condition^227,228^; 2. a neo-autoimmune or inflammatory disorder because of breaking self-tolerance; 3. a disruption of immune homeostasis in tissues; 4. a bystander self-tissue damage (on target/off tumor responses); or 5. undesired reactions to the checkpoint blockade (e.g., expression of co-inhibitory molecules on non-T cells). The most common irAEs after checkpoint blockade are dermatologic (47%–65%), colitis (30%–48%), hepatitis (5%–30%) and/or endocrine (5%–10%) with different grades of severity.^229,230^ Interestingly, irAEs are more frequent in cancer patients upon anti-CTLA-4 (60%–85%, mostly grades 1 and 2) than anti-PD-1 (16%–37%, with a minority of patients displaying high-grade toxicity) blockades.^229,231–233^ Environmental factors have been shown to influence the occurrence of irAEs. For example, the microbiome has been shown to affect efficacy and immunotoxicity of checkpoint blockade. Dysbiosis induced by antibiotics was revealed to negatively impact on the clinical outcome of cancer patients who were treated with checkpoint blockade therapies.^234–236^ Additional factors found to influence susceptibility to irAEs include the patients’ genetics/epigenetics^237^ and autoimmunity related to variants in the major histocompatibility complex (MHC) locus.^238^ A careful establishment of a risk score specific to each immunotherapy may provide important insights that can be used by clinicians to improve immunotherapeutic strategies while limiting irAEs.

## Toward tumor immunity without autoimmunity

Despite the impressive efficacy of immune checkpoint blockade in the treatment of some cancers, the manifestation of autoimmune disease-like irAEs has become a critical limitation for the applicability of these drugs in clinic. Additionally, many patients fail to respond to current checkpoint blockade therapies. Current and future efforts in the field are directed toward promoting tumor-specific immunity with greater efficacy in more clinical settings with more tolerable side effects (Fig. 2).

### Shared features of exhausted tumor-infiltrating T cells and proinflammatory CD4^+^ T helper cells

**Fig. 4:**
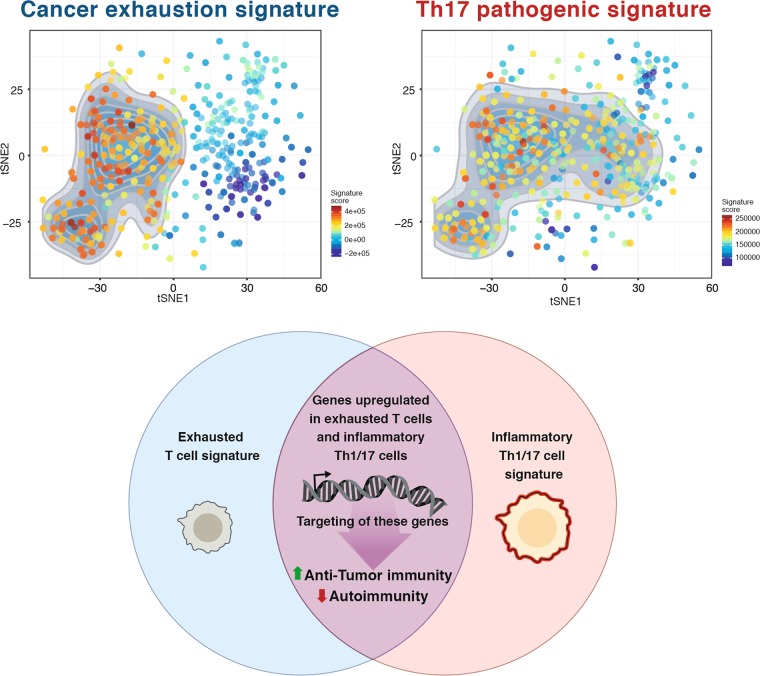
Shared transcriptional signature of inflammatory Th1 and Th17 cells and exhausted T cells. Pathogenic Th17 cells are established drivers of multiple autoimmune diseases. Exhausted T cells are hypo-functional T cells preventing active tumor immunity. Both T-cell states share part of their transcriptional signature. Interestingly, when projecting the cancer exhaustion signature^249^ and the pathogenic Th17 signature^239^ on the tSNE plot of single-cell CD8^+^ TILs (top), multiple single cells show enrichment for both signatures, suggesting that shared modules or transcriptional programs are activated in those cells. Members of this shared signature could play a key role in T-cell activation and later exhaustion. Targeting of these genes could potentially yield in an enhanced anti-tumor immunity without increased autoimmunity

As discussed in an earlier section, Th1 and Th17 cells have been implicated in the pathogenesis of multiple autoimmune diseases. Our laboratory and others have shown that Th17 cells come in multiple phenotypes.^239–241^ Th17 cells differentiated by TGF-β1 and IL-6 produce IL-17 and IL-10 and do not mediate tissue inflammation, thus are referred to as “non-pathogenic” Th17 cells.^241^ In contrast, supplementation of IL-23 to the differentiation medium leads to the generation of “pathogenic” Th17 cells.^240^ These “pathogenic” Th17 cells drive inflammation in multiple autoimmune disease mouse models.^239,242^ Using microarray analysis and single-cell RNA-seq of Th17 cells, our laboratory identified a unique transcriptional signature of pathogenic Th17 cells.^53,239^ Interestingly, when comparing the transcriptional signature of exhausted T cells to Th17 cells, it is evident that a noteworthy signature overlap exists^53,123,239^ (Fig. 4). Genes that are upregulated in exhausted T cells in cancer and in Th17 cells in autoimmunity might play an important functional role in both settings. Over the last few years, our laboratory successfully validated the functional role of some of these molecules in cancer as well as in autoimmunity. For example, the transmembrane protein podoplanin (PDPN) is highly expressed on Th17 cells and acts as a negative regulator of Th17 pathogenicity.^243^ Mice with a selective depletion of PDPN in T cells show enhanced EAE severity with increased T-cell infiltration into the CNS.^244^ Interestingly, PDPN was also identified as an important regulator in cancer. PDPN is part of the co-inhibitory gene module upregulated in cancer, and PDPN deficiency in T cells results in retardation of tumor growth.^123^ The protein C receptor (PROCR) is another molecule highly expressed in Th17 cells and exhausted T cells.^123,245^ In autoimmunity, PROCR was found to act as a negative regulator of Th17 pathogenicity.^245^ PROCR regulated the pathogenic gene module of Th17 cells by modulating expression of IL-1R, a major driver of pathogenic Th17 cells, and T cell-specific deficiency of PROCR resulted in exacerbated EAE. In the tumor setting, PROCR deficiency inhibited tumor growth with decreased frequencies in exhausted (TIM-3^High^ and PD-1^High^) CD8^+^ T cells.^123^ Another example is the glycosphingolipid receptor GPR65 that was identified in vivo to co-vary with pro-inflammatory genes in single-cell RNA-sequencing of Th17 cells during EAE.^53^ Interestingly in contrast to PDPN and PROCR, GPR65 was validated as pathogenic driver of Th17 cells in EAE. Mice deficient in GPR65 were found to be protected from EAE and showed decreased IL-17A- and IFNγ-positive cells in the spleen and lymph nodes when compared to wildtype mice. Interestingly, GPR65 was additionally identified to be part of the co-inhibitory receptor module driven by IL-27.^123^ In future studies, additional genes that are shared between proinflammatory Th1 and Th17 signatures and the exhausted T-cell signature may provide a novel set of targets that might differentially impact generation of pathogenic Th17 cells and thereby impact autoimmunity but at the same time may affect development of T-cell exhaustion and promote anti-tumor immunity (Fig. 4). Targeting these molecules has the potential to simultaneously yield benefits for tumor immunity without triggering autoimmune side-effects.

### Tumor-specific T-cell signatures

In order to target T cells specifically in the tumor, it will be important to precisely determine the differences between T cells in tumors and those at other tissue sites. Using next generation technologies (e.g., RNA-sequencing, ATAC-sequencing), it is possible to unbiasedly define tumor-specific signatures both in effector cells (CD4^+^ and CD8^+^ T cells), regulatory T cells, and myeloid cells. In a recent multi-pronged study carried out by Magnuson et al., the authors discovered a specific transcriptional profile of tumor-infiltrating Tregs across species and tumor types.^246^ This study identified multiple candidate genes encoding possible new targets for cancer immunotherapy. Using a CRISPR-based approach, the authors validated some of those targets as potential modulators of Treg infiltration and function in the tumor. The loss-of-function mutations of TNFRSF8, CXCR3, and SAMSN1 resulted in reduced frequencies of tumor-infiltrating Tregs. Similar studies combining computational, molecular, and functional systems aimed to precisely define a tumor specific CD8^+^ T-cell exhaustion gene signature in order to discover potential new targets for immunotherapies.^145,247^ A recent study identified a subpopulation of “progenitor exhausted” CD8^+^ T cells, carrying a unique genetic and epigenetic signature and being able to respond better to anti-PD-1 therapy. In melanoma patients, the frequency of these progenitor exhausted cells positively correlates with the duration of response to checkpoint-blockade therapy.^248^ These progenitors are characterized by the expression of the transcription factor TCF-1, which sustains stemness and controls effector differentiation. Interestingly, a strict antagonism between TCF-1 and TIM-3 expression exists, with TCF-1 marking stem-like progenitor cells and TIM-3 marking terminally exhausted T cells that fail to be reinvigorated by immune checkpoint blockade.^249–254^ A better understanding of this potential inverse relationship between TCF-1 and TIM-3 would be critical to manipulate T-cell state and strengthen antitumor responses upon immunotherapies. Having a clearer definition of tumor-specific T-cell phenotypes will allow the design of more tumor-restricted immunotherapies, decreasing uncontrolled responses and limiting autoimmune-like toxicity.

### Tumor-specific checkpoint blockade

An additional idea to improve checkpoint blockade is to leverage the efficacy of the current immune checkpoint blockade drugs while limiting the manifestation of irAEs at non-tumor sites. In a recent study by Perez-Ruiz et al., the authors show that prophylactic blockade of TNF ameliorates colitis but enhances tumor immunity in mice.^255^ TNF blockade is a well-established treatment option for multiple autoimmune diseases, including rheumatoid arthritis, psoriasis and IBD.^256^ Furthermore, TNF blockade is used in patients developing irAEs following immune checkpoint blockade that are refractory to steroids.^257^ In this study the authors show that the dual treatment with anti-CTLA-4 and anti-PD-1 mAbs worsens DSS-induced colitis. Interestingly, prophylactic blockade of TNF strikingly ameliorates the treatment-induced worsening of the colitis. Moreover, prophylactic TNF blockade does not hinder, but rather enhances, the anti-tumor effect of anti-CTLA-4 and anti-PD-1 combination treatment. To address potential clinical applicability, the authors further found the expression of TNF in checkpoint blockade-induced colitis to be increased in the colon when compared to healthy tissue. Based on these findings, they proposed a clinical application of TNF blockers in patients undergoing checkpoint blockade. In fact, a phase I clinical trial (https://clinicaltrials.gov; NCT03293784) is currently assessing the safety and impact on efficacy of the proposed approach. In line with the idea to optimize the “on target” aspect of checkpoint blockade, Zhang et al. designed and modified the pH-sensitivity of an anti-CTLA-4 antibody.^258^ Because of the acidic pH in the TME, one would expect the anti-CTLA-4 antibody to be preferentially active in the acidic TME. With this approach the authors were able to prevent antibody-triggered lysosomal degradation. This led to an improved immunotherapeutic effect and decreased the occurrence of irAEs in tumor-bearing mice.^258^ However, such approaches would not be effective, if the main activity of the checkpoint blockade occurs in the draining lymph nodes and not in the TME. In the future, it will be important to identify additional agents and modifications that specially inhibit the checkpoint blockade-induced autoimmunity while not impeding the anti-tumor activity.

## Conclusion

Immune checkpoint blockade recently transformed cancer treatment by showing remarkable efficacy in multiple types of cancer. However, treatment with immune checkpoint blockade is accompanied by severe autoimmune disease-like side effects that strongly limit the applicability of these drugs. As immune checkpoint blockade becomes more broadly used in cancer treatment, our understanding and treatment of these autoimmune-like irAEs need to improve. Based on our knowledge of the important function of co-inhibitory receptors in autoimmunity, immune checkpoint blockade could be improved using the basic knowledge of tissue-specific autoimmune responses and the ability of various molecules to operate in different effector T cells. Unbiased genomic approaches should be used to define tumor-specific targets for immunotherapy. Additionally, comparisons of T cells infiltrating tumors with those associated with autoimmune tissue destruction will allow identification and characterization of targets that may promote anti-tumor immunity but not drive autoimmune-like side effects. Also noteworthy is a growing body of research focused on novel tumor-restricted immunotherapy strategies beyond the scope of this review. These promising approaches include: (i) Vaccines targeting neoantigens^259–261^; (ii) Chimeric antigen receptor (CAR) T-cell therapy^262^; (iii) Improved delivery of checkpoint blockade antibodies within the TME; (iv) Bispecific antibodies^263–265^; and (v) Molecular shields restricting local activity of checkpoint inhibitors.^266^

For future research, we propose studies designed to identify novel regulatory molecules that, when targeted, simultaneously enhance anti-tumor immunity yet suppress autoimmunity.
